# New Insights into the Comprehensive System of Thermodynamic Sensors and Electronic Nose and Its Practical Applications in Dough Fermentation Monitoring

**DOI:** 10.3390/s24020352

**Published:** 2024-01-07

**Authors:** Veronika Sevcikova, Martin Adamek, Romana Sebestikova, Iva Buresova, Martin Buran, Anna Adamkova, Magdalena Zvonkova, Nela Skowronkova, Jiri Matyas, Jiri Mlcek

**Affiliations:** 1 Department of Food Analysis and Chemistry, Faculty of Technology, Tomas Bata University in Zlin, Vavreckova 5669, 760 01 Zlin, Czech Republic; v2_sevcikova@utb.cz (V.S.); aadamkova@utb.cz (A.A.); m1_zvonkova@utb.cz (M.Z.); n_skowronkova@utb.cz (N.S.); 2 Department of Automation and Control Engineering, Faculty of Applied Informatics, Tomas Bata University in Zlin, Nad Stranemi 4511, 760 05 Zlin, Czech Republic; m2adamek@utb.cz; 3 Department of Food Technology, Faculty of Technology, Tomas Bata University in Zlin, Vavreckova 5669, 760 01 Zlin, Czech Republic; r_sebestikova@utb.cz (R.S.); buresova@utb.cz (I.B.); 4 Department of Microelectronics, Faculty of Electrical Engineering and Communication, Brno University of Technology, Technicka 3058/10, 616 00 Brno, Czech Republic; martin.buran@vutbr.cz; 5 Centre of Polymer Systems, University Institute, Tomas Bata University in Zlin, Trida Tomase Bati 5678, 760 01 Zlin, Czech Republic; matyas@utb.cz

**Keywords:** thermodynamic sensors, electronic nose, rheofermentometer, dough fermentation, monitoring, mealworm flour, rice flour

## Abstract

This study focuses on an applicability of the device designed for monitoring dough fermentation. The device combines a complex system of thermodynamic sensors (TDSs) with an electronic nose (E-nose). The device’s behavior was tested in experiments with dough samples. The configuration of the sensors in the thermodynamic system was explored and their response to various positions of the heat source was investigated. When the distance of the heat source and its intensity from two thermodynamic sensors changes, the output signal of the thermodynamic system changes as well. Thus, as the distance of the heat source decreases or the intensity increases, there is a higher change in the output signal of the system. The linear trend of this change reaches an R^2^ value of 0.936. Characteristics of the doughs prepared from traditional and non-traditional flours were successfully detected using the electronic nose. To validate findings, the results of the measurements were compared with signals from the rheofermentometer Rheo F4, and the correlation between the output signals was closely monitored. The data after statistical evaluation show that the measurements using thermodynamic sensors and electronic nose directly correlate the most with the measured values of the fermenting dough volume. Pearson’s correlation coefficient for TDSs and rheofermentometer reaches up to 0.932. The E-nose signals also correlate well with dough volume development, up to 0.973. The data and their analysis provided by this study declare that the used system configuration and methods are fully usable for this type of food analysis and also could be usable in other types of food based on the controlled fermentation. The system configuration, based on the result, will be also used in future studies.

## 1. Introduction

Dough is usually prepared from wheat flour, water, yeast, and salt. Other ingredients (fat, sugar, dairy products, additives, etc.) may also be included. The creation of a bubble structure in the dough is a fundamental requirement in breadmaking. Adequate gas must be produced during fermentation; otherwise, a loaf with a low volume and hard crumb will be produced [1]. 

In the field of baking, dough quality is of paramount importance. Traditionally, sensory evaluation has been the primary method [2], but it inherently comprises subjectivity and limitations [3]. However, recent advances in dough monitoring have been realized through the integration of an E-nose and TDSs, representing significant innovations in the aforementioned field [4].

The quality of bread may be impacted by the implementation of other ingredients into the formula (wheat bran, flour of different botanical origin, insects, etc.). The presence of these ingredients may be expected to influence the production of leavening gas as well. A dough’s ability to produce and trap leavening gas may be measured using a Chopin rheofermentometer. Electronic nose (E-nose) and thermodynamic sensors (TDSs) have the potential to be cheap and useful alternatives applicable in the bread baking industry [4].

The innovation in this context lies in the combination of these technologies, which bring a comprehensive approach to dough quality assessment [4]. The E-nose, designed to mimic human olfaction, detects and quantifies volatile organic compounds (VOCs) emitted during fermentation, providing a detailed aroma and flavor profile [5]. This analytical capability not only ensures consistency but also facilitates precise control over the sensory attributes of the final product [6].

Similarly, TDSs provide data on fundamental parameters such as thermal conductivity, heat capacity, and rheological properties during dough fermentation by indirect measurement. By monitoring dough behavior, these sensors could in the future present an opportunity for bakeries to optimize processes, reduce waste and increase product consistency [6].

While these sensors were not integrated together, their parallel contributions have collectively propelled the field of dough monitoring forward. The comparative insights gleaned from E-nose and TDSs have opened new opportunities for fine-tuning and advancing our knowledge of the complexities involved in dough fermentation [4].

As we immerse into the practical applications of these sensor innovations, it becomes evident that their scientific merit transcends the conventional bounds of dough quality assessment. They serve as indispensable tools for data-driven decision making in bakery operations, allowing for real-time adjustments and improvements.

One of the most common foods is bread or other baked goods. Due to its attractiveness and widespread use, these products are enriched with natural ingredients such as edible insect flours [7] or herbs and nuts [8].

This is implemented not only to improve the nutritional properties but also to innovate standard treats [9]. For these reasons, insect flour can be used for enrichment. Insect flour is an innovative source of protein and also a modern food item [10]. For the population, insects might be more acceptable in an invisible (processed) form, as demonstrated by the sales of cricket croquettes in Belgium and The Netherlands [11,12].

It is assumed that the world’s population will reach nearly 10 billion people within 30 years. Due to the fact that conventional protein production is resource-intensive and environmentally burdensome, an alternative source of protein is being sought [13]. Standard animal farming has a high greenhouse gas production, making edible insect farming a suitable alternative protein source [14].

Food enrichment is a modern trend. Partially substituting the original ingredient with another can lead to improved nutritional properties, rheological properties of the product, and better digestibility [15].

Rice flour is suitable for enhancing the quality and digestibility of bakery products [16]. It is suitable for baking both savory and sweet baked products, and it is naturally gluten-free, making it suitable for individuals suffering from celiac disease. The use of white rice flour is versatile due to its mild taste and color. An advantage of rice flour is its higher carbohydrate content, which makes it easier to digest, and low in fat [17,18]. Its disadvantages is its low fiber content, lower protein and the fact that it does not retain much leaving gas [19,20].

The aim of this article is to explore and validate the scientific innovations represented by E-nose and TDSs; the article focuses on their transformative potential in reshaping the field of dough production. By offering cheaper, accurate and objective measurements, these sensors represent an opportunity to improve product quality in the current market. In the future, they could promote efficiency and innovation not only in the bakery industry. The main aim of the study is to validate our previous findings, which, to our best knowledge, have not been validated by any other research groups [6]. This objective was achieved not only by using other ingredients for dough preparation and by employing a different, standard baking recipe, but also through interpretation of data obtained from our experimental device and standardly used Rheofermentometer using Pearson’s correlation coefficient. Moreover, validating our previous results can become an important part for future advances in combining TDSs with E-nose and can also help us employ this experimental system in practical applications.

## 2. Materials and Methods

### 2.1. Pilot Thermodynamic Sensor Measurement

The measurement began with the pouring of water at a stable temperature, identical to the ambient temperature, with a volume of 2000 mL. After the initial stabilization of values on both sensors (approximately 1 min), 200 mL of water at 80 °C was poured into the corresponding location in the measuring container. This event represented a sudden increase in the temperature of the original water due to the pouring of heated water (as well as the cooling of the poured water). Temperature changes from this event were recorded for an additional 8 min. After completing the measurement, the water was emptied, and the same procedure was repeated for all positions (P1–P21).

### 2.2. Preparation of the Tenebrio Molitor Larvae Flour

The larvae (in the stage just before pupation; whole body length) were purchased from Radek Frýželka (Brno, Czech Republic). After purchase, the larvae were separated from the breeding substrate and starved for 48 h. They were then euthanized by immersion in boiling water at 100 °C. Subsequently, they were dried at 105 °C to a constant weight. The obtained samples were cooled (2 °C) and homogenized to approximately 1 mm particles using a coffee grinder Scarlett Silver Line SL-1545 (ARIMA, UK). The samples were stored in a plastic container in a refrigerator at 4–7 °C.

### 2.3. Ingredients and Dough Preparation

The dough was prepared according to the Rheofermentometer F4 manual [21]. This type of dough is considered the standard for bakery experiments. The dough consists of wheat refined fine flour (250 g; GoodMills a.s., Kopidlno, Czech Republic), dry yeast (3 g; Saf-instant, Lessafre group, Marcq-en-Barœul, France), and salt (5 g; K+S Czech Republic a.s., Olomouc, Czech Republic). A proportion of 5% of the wheat flour was replaced by insect flour or 5% rice flour to study the effect of these ingredients on the dough’s ability to produce and trap leavening gas. The amount of water in the formula was determined according to the flour/blend water absorption.

The ingredients were placed into the bowl of an Eta Gratus mixer (Eta, a.s., CZ, Prague, Czech Republic) and kneaded for 6 ± 1 min at 400 revolutions per minute using a dough hook. The prepared dough was divided into two parts. A weight of 315 g of dough was used to measure its ability to produce and trap leavening gas using the Rheofermentometer Rheo F4. For measurements using the TDSs and E-nose, 80 g of dough was used.

### 2.4. Measuring Using a Rheofermentometer Rheo F4

The Chopin Rheofermentometer Rheo F4 (Chopin Technologies, Villeneuve-la-Garenne, France) is a thermo-stat-controlled fermentation chamber equipped with a perforated (~0.4 mm pore) aluminum basket connected to a recorder. Dough (315 g) was placed at the bottom of the basket, pressed with a cylindrical weight (2 kg) and the chamber was hermetically closed. Fer-mentation was performed at 28 °C for 180 min. The changes in dough height, and the volume of CO_2_ produced, released and trapped in dough were recorded by an electronic microprocessor [21].

### 2.5. TDSs and E-Nose Dough Monitoring Measurements

Dough (80 g) was placed in the measuring setup of thermodynamic sensors and an electronic nose. Detailed information about the measurement system is given in Adamek et al., 2023 [6]. Dough is placed in a plastic container, with a volume of 0.2 L, and placed in a heated water bath (28 ± 2 °C). The water bath is then placed in a metal container which forms the holder of the measuring apparatus and is heated by a 12 V/5 W bulb. An automated temperature controller is used to control the water bath heating, which is controlled by the ATmega 328 microcontroller. The apparatus is located in a glass container separating the measuring apparatus from the external environment to avoid disturbing the measurements. Two thermodynamic sensors were placed in the batter and the measuring system was enclosed by a lid containing an integrated electronic nose.

Electronic nose configurations must be adapted to the nature of the individual output signals. Detecting the exact concentration is not necessary for this experiment, but it is important to detect and visualize the presence and change in quantity of each gas in an indicative manner.

Metal-oxide sensors MQ-3, MQ-8, and MQ-135 were chosen for this purpose. These sensors were selected based on previous experiences. There are of course several more types of MQ sensors but those selected cover the main substances that are released during the fermentation in this case. Another reason was easy availability in the electronic components market. The output voltage was generated by a change in the resistivity of the sensor due to the presence of a gas or substance to which the sensor was sensitive. The structure of the sensor with the sensing material was heated by the passage of an electric current through the heating element. Sensing material changes its resistivity in the presence of a monitored gas/substance. Sensor MQ-3 is particularly sensitive to alcohol, MQ-8 to hydrogen H_2_, and MQ-135 to the presence of ammonia NH_3_, alcohol, smoke, etc.

For convenience, the changes in output signals from the individual sensors were embedded into the graph in the relevant scale given by the signal from the 10-bit A/D converter, which was part of the microcontroller. The signals from the MQ-3, MQ-8, and MQ-135 sensors (Zhengzhou Winsen Electronics Technology Co., Ltd., Zhengzhou, China) were converted from a voltage level of U_out_ = 0–5 V to a digital level of d = 0–1023. In the case of the SGP30 sensor (Sensirion AG, Staefa ZH, Switzerland), the calculated TVOC signal was given in the range from 0 ppb to 60,000 ppb and the CO_2eq_ signal in the range from 400 ppm to 60,000 ppm [4,6].

The sensor carrier was firmly attached to the neck of the outer-glass container. On this carrier, the gas sensors of the electronic nose and the sensors of the thermodynamic system [22] were placed. The output signals from both devices (E-nose and TDSs) were recorded by a computer. Measurements of one sample took 180 min.

### 2.6. Statistical Analysis Methods

All data obtained from the measurements were processed and evaluated using Microsoft Excel 2019 (Microsoft Corporation, Redmond, WA, USA) and STATISTICA CZ version 12 (StatSoft, Inc., Tulsa, OK, USA).

In the case of measuring the response of the TDS signals in the water bath, the baseline of the hot water injection was shifted to U_0_ = 6 V at a time t = 60 s. Each signal was measured a total of three times. The signals were evaluated in graphical form (3D surface plot).

For the measurement of the dough properties, the curves (time series) for the TDSs and E-nose were smoothed using the moving average (m = 11), the start of the measurement was shifted to a value of U_0_ = 10 V and subsequently (in accordance with the measurement on the Rheofermentometer), the values were selected in multiples of 90 s. This step was necessary to ensure a follow-up comparison of the time series. The time series were compared using Pearson’s correlation coefficient.

Significant differences between samples were determined by analysis of variance, considering significant differences (*p* < 0.05). The Shapiro–Wilk test of normality, Levene’s test of homogeneity, and Brown–Forsythe tests were performed for all monitored samples. If any of the assumption tests were not successful (marked as X), the result could not be decided based on the ANOVA analysis. Therefore, the non-parametric tests Kruskal–Wallis one-way analysis of variance (α = 0.05) and the Median test (α = 0.05) had to be performed.

## 3. Results and Discussion

### 3.1. Description of Thermodynamic Sensor Positioning and Their Response to the Signal Source

This subsection builds upon previous measurements. The motivation for the experiment was to expand and refine the analysis of the mutual position of sensors and their sensitivity to temporal temperature impulses. The collected and evaluated data can not only help in gaining a deeper understanding of heat exchange within a substance but also raise further questions about the entire phenomenon [4,6].

**Figure 1:**
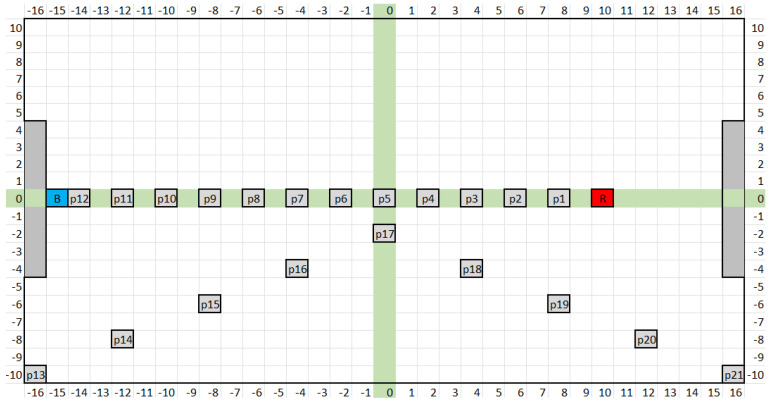
Sensor location map (red—TDS1 and blue—TDS2) in the plastic container and individual positions (P1–P21) for refilling heated water. The values on the X and Y lines correspond to the distances from the center of the vessel in centimeters.

In this experiment, measurements were performed in a plastic container with a water bath at a temperature of 23 °C, into which the thermodynamic system sensors TDS1 and TDS2 were inserted at fixed positions. Subsequently, the response of the TDSs to the addition of hot water (80 °C) at the selected positions in the water bath was monitored. Positions P1–P12 were designed on the direct line between the TDS1 and TDS2 sensors (along the horizontal axis in Figure 1). Positions P13–P21 were designed on diagonal axes at different distances from both sensors (see Figure 1).

The map in Figure 1 shows the location of the sensors (red—TDS1, and blue—TDS2) in the plastic container and the individual positions (P1–P21) for adding heated water. The values on the X and Y lines correspond to the distances from the center of the vessel in centimeters.

After 60 s of settling, hot water was poured onto the position. This event represented a sudden increase in the temperature of the original water due to the spilling of the heated water (as well as the cooling of the poured water). Changes in the TDS output response as a function of the temperature change from this event were recorded for an additional 8 min. After the measurements were completed, the water was discharged. The same procedure was repeated for all positions (P1–P21). The entire measurement process was repeated a total of three times due to time consumption.

**Figure 2:**
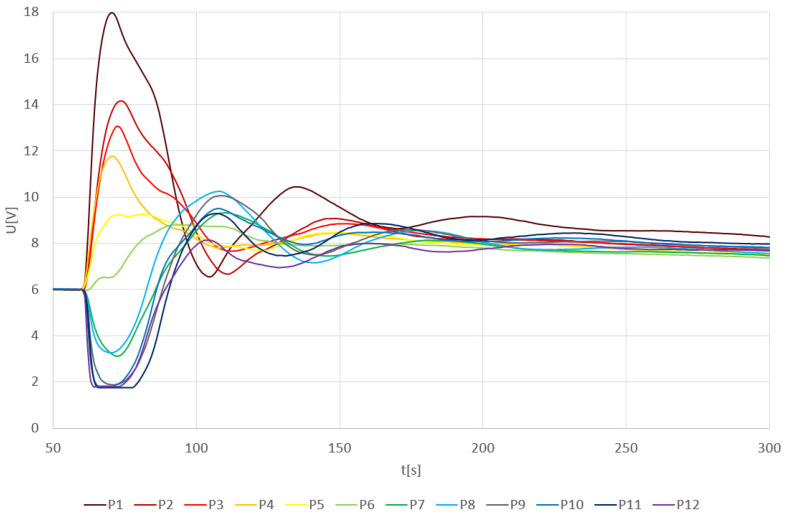
Output voltage U from the TDS system depending on the average value of heat pulses and P1—P12 positions.

The results of an arithmetical average of individual thermal pulses are shown in a 2D plot (Figure 2). The measurement range and the operating range of the measuring circuit for this case were from 1.8 V to 18 V.

The results prove that the closer the pulse source is to sensor TDS1 (resulting in a more intense thermal shock), the larger the response of the measuring circuit. The most significant increase in the output signal was recorded at point P1. As the distance from this point increases, the positive voltage becomes smaller (P2–P8). Beyond a certain point, from P9 onwards up to P12, the trend reverses. The voltage starts to increase into negative values due to the thermal shock on the sensor TDS1. However, through mixing and stabilizing the temperature of the water solution over time, the output signal stabilizes at the same values as for other positions.

**Figure 3:**
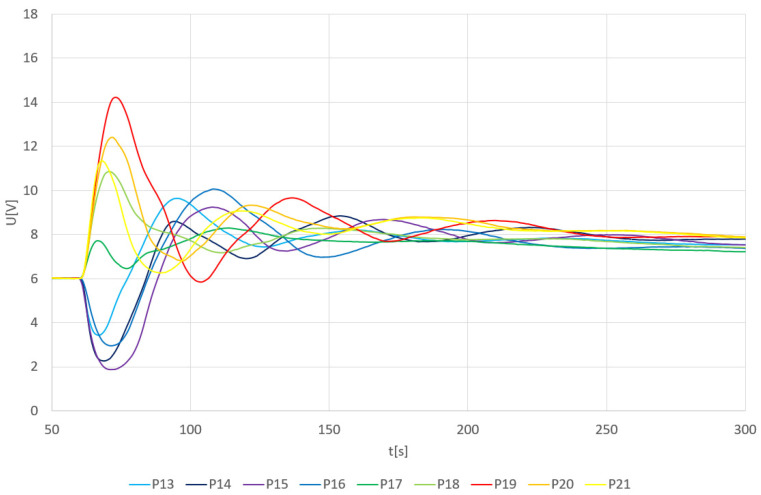
Output voltage U from the TDS system depending on the average value of heat pulses and P13–P21 positions.

**Figure 4:**
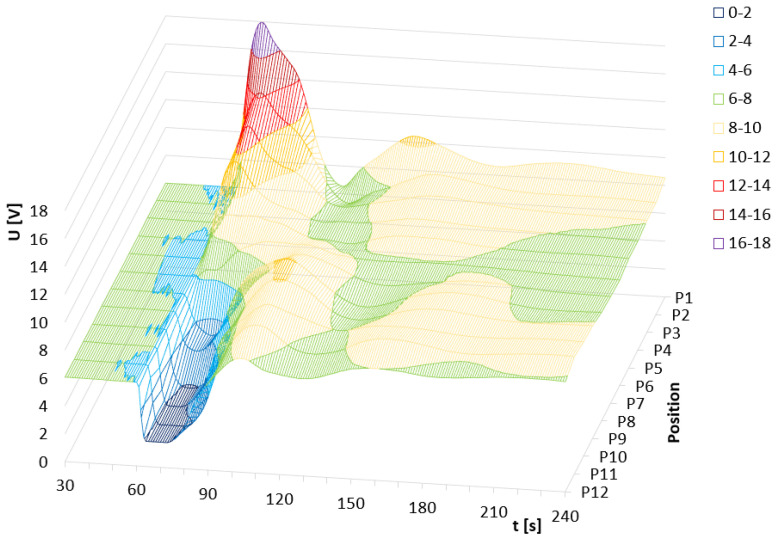
The output response of the TDS system depending on the average value of heat pulses and P1–P12 positions. For positions P10–P12, the values of 1 peak hit the limit of the power supply.

The output response of the TDS system depending on the individual heat pulses in positions P13–P21 is shown in Figure 3. In the proximity of the TDS2 sensor (positions P13–P16), the first peak signal goes below the initial level again. However, due to the larger distance from TDS1, the output signal is not limited by the voltage source. As the position gets closer to TDS1, the first peak rises again above the initialization level. Due to the greater distance of the positions (especially P18–P20) from the TDS1 sensor, the maximum peak height is lower compared to the graph in Figure 4. Interestingly, the peak height value at position P21 is comparable to the maximum peak values even though the position is already relatively far from the TDS1 sensor.

**Figure 5:**
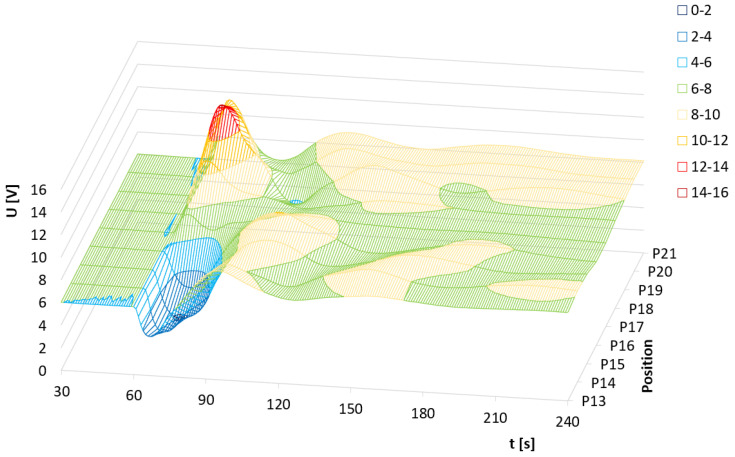
TDS output response depending on the average value of heat pulses and positions P13–P21.

Another observable effect is the increase in the voltage level, from the state before the thermal pulse to the voltage level after the thermal pulse has stabilized. This voltage increase corresponds to the change in water temperature according to the general calorimetric description published in Adamek et al., 2023 [6]. The previous data and findings presented in Figure 2 and Figure 3 can be also visible in its 3D representation presented in Figure 4 and Figure 5.

**Figure 6:**
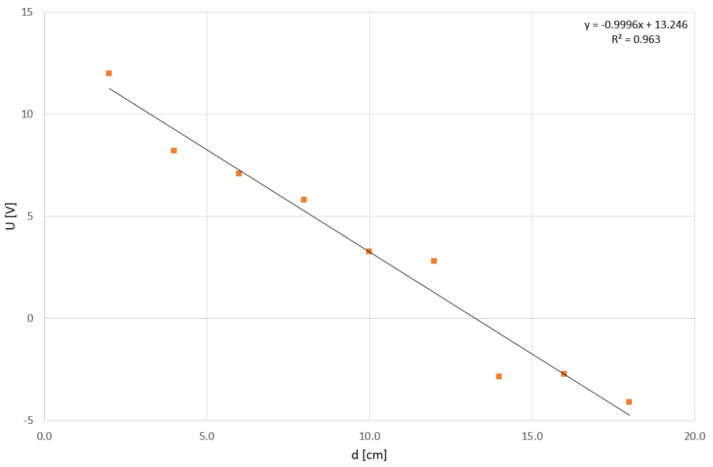
Dependence of the first-peak output voltage on direct distance from TDS Red (positions 1–9).

Another perspective on the system’s response can be obtained by comparing the maximal values of individual pulses relative to their position. As evident from the previous results, the output voltage U decreases with the increasing distance of the thermal pulse from the TDS Red sensor. The trend of this change is illustrated in Figure 6.

Figure 6 describes the maximum values of the first peak, both positive and negative, immediately after pouring hot water (80 °C). However, in some curves, the first peak may not be the one with the maximum voltage value U. The voltage value of 0 V in Figure 6 corresponds to the 6 V value in Figure 2 and Figure 3 at 60 s. Therefore, an offset of −6 V is applied here.

The points, each representing the peak value for a specific position, are fitted with a linear trend line with an R^2^ value of 0.963. Only measurements taken from positions 1–9 were used for this comparison.

**Figure 7:**
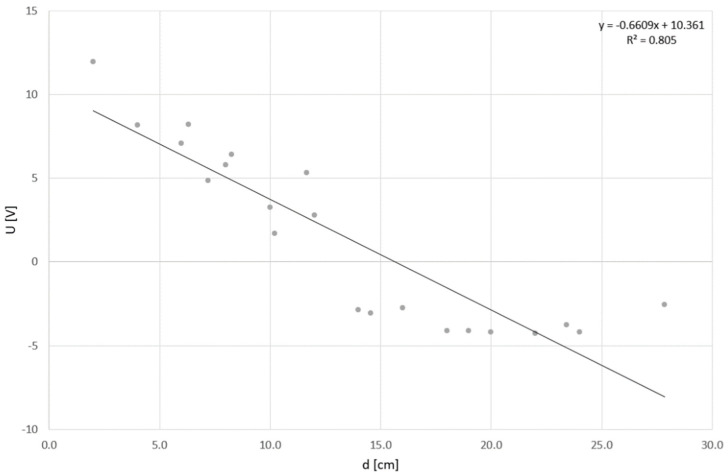
Dependence of the first-peak output voltage on direct distance from TDS Red (all positions).

The rest of the positions are the farthest from the TDS Red sensor resp. they are in an indirect position relative to the TDS Red-Blue sensors. Their inclusion reduces the reliability coefficient of the linear function to an R^2^ value of 0.805. This can be seen in Figure 7.

The behavior of the system in the nonlinear part of the measurement spectrum has not yet been fully clarified. Clarifying the behavior of this anomaly could involve increasing the number of measuring points in that area or utilizing additional supporting measuring instruments. A heat flow sensor, similar to thermodynamic sensors, can be used for measuring heat flows. Technology based on the printed circuit board is one of the possible alternatives. Different designs can bring higher measurement accuracy due to less thermal resistance and other improved properties [23]. Their specific design could also be employed for direct monitoring of heat flows at the edge of the entire measuring system [24].

Alternatively, it is possible to entirely modify the experimental methodology and employ calorimetric methods in a suitable configuration. For an exact measurement of heat consumption and heat exchange measurement, using calorimetric methods will be much more precise [25].

For measurement in the dough fermentation process, the thermodynamic sensor methods are suitable enough, as can be seen in the following sections.

### 3.2. Dough Fermentation Monitoring and Comparison of the Measurement Results

The following experiment was focused on the monitoring of dough fermentation using TDS, experimental E-nose, and rheofermentometer. The measurements were carried out with wheat flour without and with the addition of 5% rice flour and with 5% insect meal. The paper mainly focuses on the statistical results of the different methods of comparison.

Several authors have suggested enriching the dough with insect meal due to the rheological properties of the dough. This parameter significantly impacts the dough volume because it helps keep rising gas inside the dough volume Kowalski et al., 2022 [7], González et al., 2019 [10] and Cappeli et al., 2020 [26]. In a previous study conducted by Adamek et al., 2023 [5] the same methodology was used. The results show that the highest correlation of results between the rheofermentometer and E-nose was for dough enriched with 5% insect meal; therefore, the authors focused on this fortification amount.

Rice flour was added to measure the rheological changes of the dough. When rice flour was incorporated, a significant increase in swelling power and bulk density in the flour blend was observed, while a significant decrease in oil absorption capacity occurred. These findings are reported by Jan et al., 2022 [16] in their study. A study by Sabanis 2007 [27] supplemented bread wheat flour with rice, corn, and soy. Doughs produced by supplementation up to 10% had satisfactory rheological properties and the bread had acceptable quality attributes (color, taste, and flavor).

**Figure 8:**
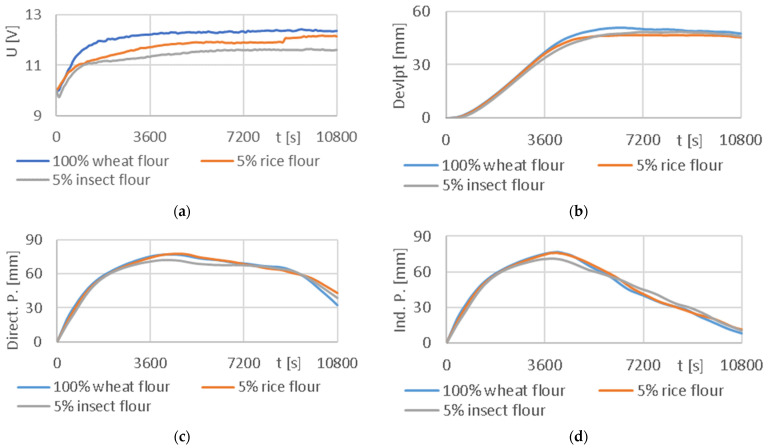
Comparison of the output signal U from the experimental TDSs with the output signals from the rheofermentometer: (**a**) output signal U [V] from the TDSs; (**b**) dough development curve [mm]; (**c**) gas release curve—Total volume; (**d**) gas release curve—retention volume.

**Figure 9:**
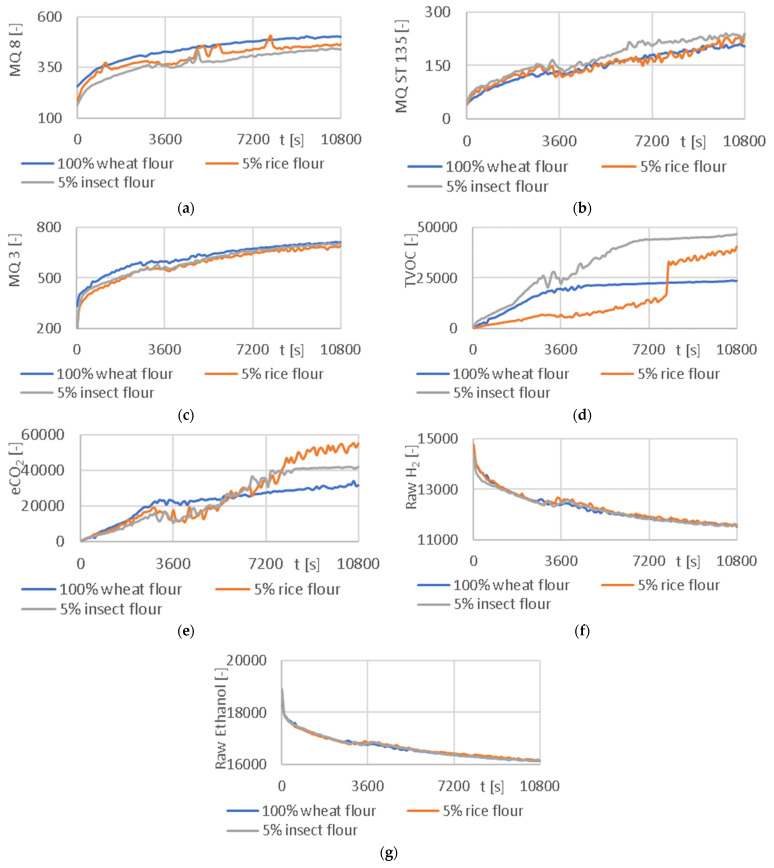
Signal (average values) for different types of flour mixtures and individual E-nose sensors: (**a**) output signal from MQ 8 sensor; (**b**) output signal from MQ ST 135 sensor; (**c**) output signal from MQ 3 sensor; (**d**) output signal from TVOC sensor; (**e**) output signal from eCO_2_ sensor; (**f**) output signal from Raw H_2_ sensor; (**g**) output signal from Raw Ethanol sensor.

According to the methodology, the prepared dough was divided into two parts. The first part was put into a rheofermentometer, from which signals were obtained to change the volume of the developed dough referred to as Devlpt, the amount of gas formed—Direct. P. and the amount of gas released from the dough—Ind. P. The second portion of the prepared dough was placed in an experimental apparatus consisting of a TDS thermodynamic system and an experimental electronic nose (described above). The TDS system and sensor output response from the electronic nose was recorded and processed. The resulting curves of the average values from each of the measured flour types for the signals from the rheofermentometer and the TDS system are shown in Figure 8 and for the signals from the electronic nose in Figure 9.

The addition of rice and insect flours decreased the values of output signal U obtained by the experimental TDSs (Figure 8a). The impact on the characteristics recorded by the rheofermentometer was weak. However, the negative impact of the presence of insect flour on dough volume (Figure 8b), and the amount of produced (Figure 8c), and released gas (Figure 8d) was observed.

A comparison of the time series from the TDSs and the variables obtained from the rheofermentometer using Pearson’s correlation coefficient is shown in Table 1. The table documents the highest values of the correlation coefficient for the dough volume development signal (Devlpt), where it reached up to 0.932. On the other hand, the lowest correlation found was for the volume of gas retained in the dough (Ind. P.), where for the mixture of 95% wheat + 5% rice flour, the value dropped to 0.060 and the correlation was rated as insignificant. The column Direct. P. shows the correlation of TDSs with the amount of gas produced. These values ranged from 0.707 to 0.785 and were assessed as statistically significant.

This paper’s results thus specify the results from Adamek (2023) [6] and show that the signal from the TDS system correlates most closely with dough volume development. Although the original results of the correlation between TDSs and Ind. P. ranged from −0.853 to +0.879 and some appeared to be statistically significant, the low value of the correlation coefficient was confirmed after refinement. In the case of a mixture of 95% wheat flour and 5% rice flour, a statistically insignificant value of 0.060 was even determined.

Table 2 contains the correlation coefficients calculated by comparing the signals from the rheofermentometer and the sensors from the experimental electronic nose. The signals from the E-nose sensors correlated well with the volume of dough developed (Devlpt), where the TVOC sensor achieved a result of up to 0.973 for 100% wheat flour and 0.968 for a mixture of 95% wheat + 5% insect flour. The highest average value of 0.922 of the correlation coefficients for all measured flours in each sensor was found for the MQ 3 sensor detecting mainly alcohol. The sensors detecting hydrogen H_2_ had a similar average value (MQ 8—0.915; Raw H_2_—−0.916).

The course of these reactions was more turbulent. In contrast, a previous study found that TDSs and E-nose data correlated most with the amount of gas and least with the volume of dough, forming the exact opposite. In a previous study by Adamek et al., 2023 [6], pure gluten-free dough with the addition of insect meal was measured; since our study focused on flours containing gluten, these different results are likely due to this factor. Anton and Arfied, 2007 [28] reported differences between the rheological properties of standard wheat and gluten-free doughs.

All the results obtained from the tested positions (P1–P21) of the heat source can significantly contribute to the construction of special containers for dough fermentation, especially in the bakery industry, when it is necessary to control the exact conditions of fermentation for certain doughs. Seven sensors of the electric nose bring comprehensive and precise information, which is very important in connection with the positions of the heat source, and thus it is possible to more thoroughly monitor doughs with unusual ingredients, where the fermentation process is not entirely predictable.

To complete the comprehensive evaluation of dough parameters, a comparison of individual parameters (Table 3) characterizing the properties of the dough during its rising, measured using a rheofermentometer Rheo F-4 device (Chopin, France), is presented. The results confirmed a statistically significant difference in the parameter Hm (maximum development reached by the dough, correlated with bread volume). For the parameters T1 (time required for maximum development, in relation to yeast activity), Tx (time of appearance of porosity in the dough), and h (the height of the dough at the end of the measurement) the result of the differences cannot be decided, and further measurement is required. For other parameters ((Hm − h)/Hm—decline in dough development, H’m—maximum height of the gas curve, T’1—time required to reach H’m, Vt—total volume of CO_2_, Vr—volume of retention CO_2_, Vc—released CO_2_ volume, Vr/Vt—gas retention coefficient), no statistically significant difference was detected.

## 4. Conclusions

This study and the conducted experiments followed up on previous studies carried out in the development of a complex system of electronic nose and thermodynamic sensors.

First of all, the measurements themselves were carried out to gain a deeper understanding of the behavior of the thermodynamic sensor system. In this phase, it was validated that the distance of the heat source and its intensity from the sensors has a direct influence on the output signal value of the sensor system. Thus, it can be concluded that as the distance of the heat source decreases or the intensity increases, there is a greater change in the output signal of the system. The previous statement is supported by mathematical analysis. Peak voltage values fitted with a linear trend line reached an R^2^ value of 0.963. If we include data farthest from TDSs, and data in an indirect position, respectively, the value of R^2^ reached 0.805. An accurate representation of this dependence in the two-dimensional domain along with the measured data is provided in the results section. Confirmation of linear dependency is key to obtaining the highest signal response during TDSs measurements. The source of the main signal response is located directly between both measuring sensors. Therefore, we will keep this sensor positioning and recommend it for all future measurements.

Furthermore, the method of the complex measurement of wheat dough fermentation with different ingredients added into the standard mixture was validated. Mealworm flour and rice flour were added at 5% to standard wheat dough, which was measured by thermodynamic sensors and an electronic nose. Measurements were also made using a rheofermentometer and statistical evaluation of data correlation was performed. The results show that the data obtained by the thermodynamic sensors and the electronic nose correlated strongly with the volume of dough formed. Pearson´s correlation coefficient for the TDSs and rheofermentometer reached up to 0.932 for dough volume development. Also, the E-nose signals correlated well with the dough volume development, up to 0.973 in the case of the TVOC sensor. This correlation was also high for the alcohol and hydrogen sensors, measuring 0.915 and 0.916.

All the presented results prove that the equipment and methods used in the given configuration can be used not only for measuring the mentioned types of dough but also others, or can be applied for other foods where changes occur during fermentation.

## Figures and Tables

**Table 1:** Correlation coefficient when comparing the signal from the TDSs with the developed dough volume change (Devlpt), amount of formed gas (Direct. P.), and amount of released gas (Ind. P.).

Type of Flour	Devlpt	Direct. P.	Ind. P.
100% wheat	0.821609	0.777774	0.242257
95% wheat + 5% rice	0.932442	0.707197	0.060490
95% wheat + 5% insect flour	0.885963	0.785930	0.197879

**Table 2:** Correlation coefficient when comparing the signal from the experimental E-nose sensors with developed dough volume change (Devlpt), amount of formed gas (Direct. P.), and amount of released gas (Ind. P.).

Sensor	Type of Flour	Devlpt	Direct. P.	Ind. P.
MQ 8 (H_2_)	100% wheat	0.942692	0.531024	−0.154068
	95% wheat + 5% rice	0.880930	0.582673	−0.105621
	95% wheat + 5% insect meal	0.920672	0.624977	−0.049346
MQ ST 135 (NH_3_)	100% wheat	0.897375	0.366279	−0.334604
	95% wheat + 5% rice	0.815331	0.356613	−0.336980
	95% wheat + 5% insect meal	0.904386	0.465991	−0.250637
MQ 3 (alcohol)	100% wheat	0.921239	0.511421	−0.171955
	95% wheat + 5% rice	0.922859	0.590863	−0.119926
	95% wheat + 5% insect meal	0.921120	0.582981	−0.103838
TVOC	100% wheat	0.972722	0.654838	0.027287
	95% wheat + 5% rice	0.607699	0.009739	−0.630009
	95% wheat + 5% insect meal	0.967926	0.542631	−0.154611
eCO_2_	100% wheat	0.946134	0.533073	−0.124684
	95% wheat + 5% rice	0.734810	0.162583	−0.550390
	95% wheat + 5% insect meal	0.858553	0.328447	−0.405467
Raw H_2_	100% wheat	−0.931882	−0.517683	0.167196
	95% wheat + 5% rice	−0.895538	−0.564956	0.143315
	95% wheat + 5% insect meal	−0.920743	−0.562974	0.137113
Raw Ethanol	100% wheat	−0.919482	−0.507990	0.172027
	95% wheat + 5% rice	−0.886267	−0.577300	0.118399
	95% wheat + 5% insect meal	−0.911905	−0.609291	0.067512

**Table 3:** Results of statistical differences between doughs using the rheofermentometer Rheo F-4 device (Chopin, France).

Parameters	Tests						
	S-W ^1^	Levene ^2^	B-F ^3^	ANOVA ^4^	K-W ANOVA ^5^	Median ^6^	Difference
Dough development curve							
Hm [mm]	**X**	OK	OK	**0.003**	**0.038**	**0.043**	Difference confirmed.
h [mm]	**X**	**X**	OK	**0.018**	0.061	**0.043**	Cannot decide on the difference
(Hm − h)/Hm [%]	OK	OK	OK	0.259			There is no difference
T1 [min]	**X**	**X**	OK	0.417	0.298	**0.043**	Cannot decide on the difference
Gas release curve							
H’m [mm]	OK	**X**	OK	0.081	0.177	0.165	There is no difference
T’1	OK	OK	OK	0.133			There is no difference
Tx	**X**	OK	OK	**0.037**	0.070	**0.043**	Cannot decide on the difference
Vt [mL] (Total)	OK	OK	OK	0.132			There is no difference
Vr [mL] (Retention)	OK	OK	OK	0.066			There is no difference
Vc [mL] (CO_2_)	**X**	OK	OK	0.245	0.252	0.638	There is no difference
Vr/Vt [%] (CR)	OK	OK	OK	0.301			There is no difference

^1^ Shapiro–Wilk normality test, ^2^ Levene Test for Equality of Variances, ^3^ Brown–Forsythe test, ^4^ analysis of variance, ^5^ Kruskal–Wallis one-way analysis of variance, and ^6^ Median test. Bold numbers or a bold letter X indicate tests where a statistically significant difference was found.

## Data Availability

New research data were presented in this contribution.

## References

[1] Kent N.L., Evers A.D. (1994). Bread-baking Technology. Technology of Cereals.

[2] Duizer L.M., Walker S.B. (2016). The Application of Sensory Science to the Evaluation of Grain-Based Foods. Encyclopedia of Food Grains.

[3] Shu N., Chen X., Sun X., Cao X., Liu Y., Xu Y.J. (2022). Metabolomics identify landscape of food sensory properties. Crit. Rev. Food Sci. Nutr..

[4] Adamek M., Adamkova A., Mlcek J., Vojackova K., Famera O., Buran M., Hlobilova V., Buckova M., Baron M., Sochor J. (2020). Sensor systems for detecting dough properties fortified with grape pomace and mealworm powders. Sensors.

[5] Tan J., Xu J. (2020). Applications of electronic nose (e-nose) and electronic tongue (e-tongue) in food quality-related properties determination: A review. Artif. Intell. Agric..

[6] Adamek M., Zvonkova M., Buresova I., Buran M., Sevcikova V., Sebestikova R., Adamkova A., Skowronkova N., Mlcek J. (2023). Use of a Thermodynamic Sensor in Monitoring Fermentation Processes in Gluten-Free Dough Proofing. Sensors.

[7] Kowalski S., Mikulec A., Mickowska B., Skotnicka M., Mazurek A. (2022). Wheat bread supplementation with various edible insect flours. Influence of chemical composition on nutritional and technological aspects. LWT.

[8] Vishwakarma S., Dalbhagat C.G., Mandliya S., Mishra H.N. (2022). Investigation of natural food fortificants for improving various properties of fortified foods: A review. Food Res. Int..

[9] Ibrahim U.K., Salleh R.M., Maqsood-ul-Haque S.N.S. (2015). Bread towards functional food: An Overview. ETP Int. J. Food Eng..

[10] González C.M., Garzón R., Rosell C.M. (2019). Insects as ingredients for bakery goods. A comparison study of *H. illucens*, *A. domestica* and *T. molitor* flours. Innov. Food Sci. Emerg. Technol..

[11] Poma G., Cuykx M., Amato E., Calaprice C., Focant J.F., Covaci A. (2017). Evaluation of hazardous chemicals in edible insects and insect-based food intended for human consumption. Food Chem. Toxicol..

[12] Stoops J., Vandeweyer D., Crauwels S., Verreth C., Boeckx H., Van Der Borght M., Claes J., Lievens B., Van Campenhout L. (2017). Minced meat-like products from mealworm larvae (*Tenebrio molitor* and *Alphitobius diaperinus*): Microbial dynamics during production and storage. Innov. Food Sci. Emerg. Technol..

[13] Probst L., Frideres L., Pedersen B., Amato F. (2015). Safe and nutritious food new nutrient sources. Business Innovation Observatory Contract No 190/PP/ENT/CIP/12/C/N03C01.

[14] Ordoñez-Araque R., Quishpillo-Miranda N., Ramos-Guerrero L. (2022). Edible Insects for Humans and Animals: Nutritional Composition and an Option for Mitigating Environmental Damage. Insects.

[15] Osendarp S.J.M., Martinez H., Garrett G.S., Neufeld L.M., De-Regil L.m., Vossenaar M., Darnton-Hill I. (2018). Large-Scale Food Fortification and Biofortification in Lowand Middle-Income Countries: A Review of Programs, Trends, Challenges, and Evidence Gaps. Food Nutr. Bull..

[16] Jan N., Naik H.R., Gani G., Bashir O., Amin T., Wani S.M., Sofi S.A. (2022). Influence of replacement of wheat flour by rice flour on rheo-structural changes, in vitro starch digestibility and consumer acceptability of low-gluten pretzels. Food Prod. Process Nutr..

[17] Juliano B.O. (1993). Rice in Human Nutrition.

[18] Gujral H.S., Rosell C.M. (2004). Improvement of the breadmaking quality of rice flour by glucose oxidase. Food Res. Int..

[19] Luh B.S., Liu Y.K., Luh B.S. (1991). Rice Flours in Baking. Rice.

[20] Buresova I., Cervenka L., Sebestikova R., Augustova M., Jarosová A. (2023). Applicability of Flours from Pigmented and Glutinous Rice in Gluten-Free Bread Baking. Foods.

[21] Chopin Technologies (2016). Rheo F4 User’s Manual 12/2016.

[22] Adamek M., Matyas J., Adamkova A., Mlcek J., Buran M., Cernekova M., Sevcikova V., Zvonkova M., Slobodian P., Olejnik R. (2022). A Study on the Applicability of Thermodynamic Sensors in Fermentation Processes in Selected Foods. Sensors.

[23] Ozaki Y., Matsui H., Shimizu M. (2018). Heat flow sensor created by printed circuit board manufacturing processes. Proceedings of the International Conference on Electronics Packaging and iMAPS All Asia Conference (ICEP-IAAC).

[24] Hukseflux Thermal Sensors. FHF05 Series Heat Flux Sensors. https://www.hukseflux.com/products/heat-flux-sensors/heat-flux-sensors/fhf05-series-heat-flux-sensors.

[25] Fiock E.F. (2023). A Review of Calorimetric Measurements on Thermal Properties of Saturated Water and Steam. ASME Trans..

[26] Cappelli A., Oliva N., Bonaccorsi G., Lorini C., Cini E. (2020). Assessment of the rheological properties and bread characteristics obtained by innovative protein sources (*Cicer arietinum*, *Acheta domesticus*, *Tenebrio molitor*): Novel food or potential improvers for wheat flour?. LWT Food Sci. Technol..

[27] Sabanis D., Tzia C. (2009). Effect of Rice, Corn and Soy Flour Addition on Characteristics of Bread Produced from Different Wheat Cultivars. Food Bioprocess Technol..

[28] Anton A.A., Artfield S.D. (2007). Hydrocolloids in gluten-free breads. A review. Int. J. Food Sci. Nutr..

